# Association between ecological factors and emergency care visits: The influence of relative humidity

**DOI:** 10.1192/j.eurpsy.2021.1878

**Published:** 2021-08-13

**Authors:** D. Hernández-Calle, M.F. Bravo-Ortiz

**Affiliations:** Psychiatry, Hospital Universitario La Paz, Madrid, Spain

**Keywords:** Emergency Care, Ecological factors

## Abstract

**Introduction:**

Psychiatric emergency visits have been associated to several climate variables. However, the influence of relative humidity has been not well stablished.

**Objectives:**

The analyse the influence of relative humidity on emergency care visits.

**Methods:**

Daily urgency visits were extracted from electronic medical records of Hospital Universitario La Paz from 1st January 2019 to 31st December 2019. Relative humidity data (%) was obtained from a local climate station. A negative binomial multivariate regression model was performed with relative humidity, weekday and month as covariates.

**Results:**

Relative humidity was not associated with number of psychiatric emergency department visits (IRR 1.00; 95%CI 0.99-1.00)

**Conclusions:**

Relative humidity did not influence emergency help seeking for patients suffering from suicidal phenomena

**Disclosure:**

No significant relationships.

